# Epigenetic Landscape in Pancreatic Ductal Adenocarcinoma: On the Way to Overcoming Drug Resistance?

**DOI:** 10.3390/ijms21114091

**Published:** 2020-06-08

**Authors:** Sona Ciernikova, Julie Earl, María Laura García Bermejo, Viola Stevurkova, Alfredo Carrato, Bozena Smolkova

**Affiliations:** 1 Department of Genetics, Cancer Research Institute, Biomedical Research Center of the Slovak Academy of Sciences, Dúbravská cesta 9, 845 05 Bratislava, Slovakia; viola.stevurkova@savba.sk; 2 Molecular Epidemiology and Predictive Tumor Markers Group, Medical Oncology Research Laboratory, Ramón y Cajal Health Research Institute (IRYCIS), Carretera Colmenar Km 9100, 28034 Madrid, Spain; julie.earl@live.co.uk (J.E.); acarrato@telefonica.net (A.C.); 3 Biomarkers and Therapeutic Targets Group, Ramón y Cajal Health Research Institute (IRYCIS), Carretera Colmenar Km 9100, 28034 Madrid, Spain; garciabermejo@gmail.com; 4 Department of Molecular Oncology, Cancer Research Institute, Biomedical Research Center of the Slovak Academy of Sciences, Dúbravská cesta 9, 845 05 Bratislava, Slovakia; bozena.smolkova@savba.sk

**Keywords:** pancreatic ductal adenocarcinoma, drug resistance, epigenetics, epithelial–mesenchymal transition, liquid biopsy, epi-drugs

## Abstract

Pancreatic ductal adenocarcinoma (PDAC) is one of the most aggressive solid malignancies due to the rapid rate of metastasis and high resistance to currently applied cancer therapies. The complex mechanism underlying the development and progression of PDAC includes interactions between genomic, epigenomic, and signaling pathway alterations. In this review, we summarize the current research findings on the deregulation of epigenetic mechanisms in PDAC and the influence of the epigenome on the dynamics of the gene expression changes underlying epithelial–mesenchymal transition (EMT), which is responsible for the invasive phenotype of cancer cells and, therefore, their metastatic potential. More importantly, we provide an overview of the studies that uncover potentially actionable pathways. These studies provide a scientific basis to test epigenetic drug efficacy in synergy with other anticancer therapies in future clinical trials, in order to reverse acquired therapy resistance. Thus, epigenomics has the potential to generate relevant new knowledge of both a biological and clinical impact. Moreover, the potential, hurdles, and challenges of predictive biomarker discoveries will be discussed, with a special focus on the promise of liquid biopsies.

## 1. Introduction

According to GLOBOCAN data, pancreatic cancer is ranked the 11th most common cancer worldwide, being the 7th leading cause of global cancer deaths in industrialized countries. Strikingly, the number of deaths from pancreatic cancer in 2018 (432,000) was very close to the number of new cases (459,000) [1]. The incidence of the disease is almost the same for both sexes and significantly increases with age, while the average age at diagnosis is 71 years [2]. The early stages usually lack symptoms, leading to a late diagnosis (stages III or IV) with limited therapeutic options and a poor prognosis. Consequently, the absence of biomarkers for early-stage detection makes this oncological disease one of the most lethal. By 2030, it is projected to be the second leading cause of cancer-related death, even surpassing breast, prostate, and colorectal cancer [3]. In Europe, there were 132,560 pancreatic cancer deaths in 2018; the Slovak Republic is ranked third in incidence after Lithuania and Estonia and fourth in mortality with 1040 cases, after Malta, the Czech Republic, and Hungary [4].

**Figure 1:**
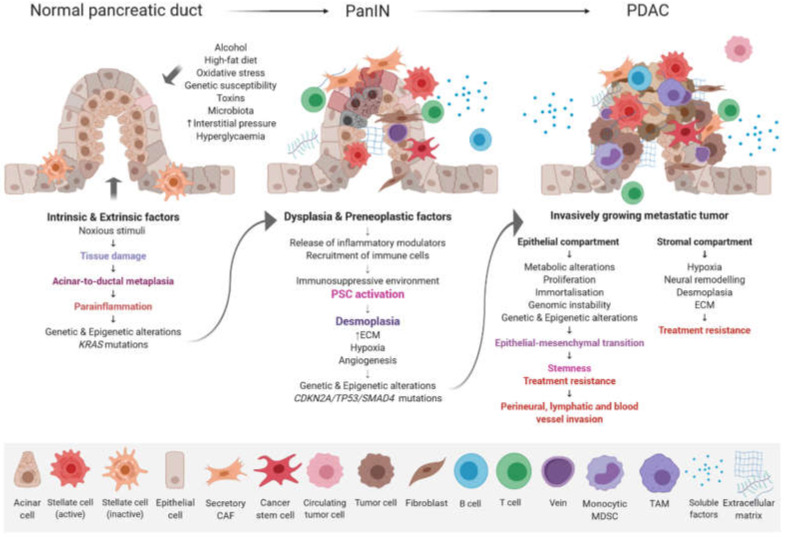
Progression model of PDAC. The stepwise accumulation of morphologic, histopathologic, genetic, and epigenetic changes are accompanied by immune cell infiltration and a desmoplastic stromal reaction. Abbreviations: CAF, cancer-associated fibroblast; ECM, extracellular matrix; MDSC, myeloid-derived suppressor cell; PSC, pancreatic stellate cell; TAM, tumor-associated macrophage.

The possible risk factors include gender, age, smoking, alcohol abuse, obesity, a lack of physical activity, diabetes, chronic pancreatitis, vitamin D deficiency, genetic susceptibility, as well as dietary and reproductive causes [5]. Overall, the accumulation of genetic, epigenetic, molecular, and morphological changes in pancreatic ductal cells causes disease initiation and development. Three non-invasive pancreatic neoplasia differing in biological and clinical behavior have been described, namely intraductal papillary mucinous neoplasm (IPMN), mucinous cystic neoplasm (MCN), and pancreatic intraepithelial neoplasia (PanIN). PanIN is divided into four stages, i.e., Pan IN-1A, Pan IN-1B, Pan IN-2, and Pan IN-3, each being associated with specific molecular alterations [6]. The development of pancreatic ductal adenocarcinoma (PDAC) involves the progression from hyperplasia through to dysplasia and finally to a malignant state [7] (Figure 1).

PDAC arises in the exocrine glands of the pancreas, although some acinar cells are highly flexible and can experience acinar to ductal metaplasia under stress conditions, and accounts for 95% of all pancreatic cancers. A small group of exocrine tumors, which account for only 1%, include acinar cell carcinomas, solid pseudopapillary tumors, and pancreatoblastoma. These mainly occur in pediatric patients. The last group consists of neuroendocrine tumors (PanNETs) that occur in the endocrine tissue and comprise less than 5% of all cases [8]. The metastatic potential of PDAC is extremely high and tumors spread mainly through lymphatic and blood vessels. Most of the patients already have metastasis in the liver and lymph nodes at the time of diagnosis [9]. According to epidemiological data, less than 20% of cases survive one year and the five-year survival rate remains around 5–7% [10]. Despite intensive research, surgical resection of the pancreas with microscopically free margins (R0 resection) remains the only realistic and potentially curative option. Unfortunately, rapid disease progression and early metastasis are responsible for the late diagnosis and less than 20% of patients have a resectable tumor at the time of diagnosis [11]. Moreover, the wide heterogeneity of genetic mutations and dense stromal microenvironment make PDAC one of the most drug-resistant cancers [12]. This, together with the lack of an effective therapy, leads to a dismal prognosis of PDAC patients.

Although genetic modifications are well defined in PDAC, the role of epigenetics regulations, which secure dynamic response to environmental stimuli, have only recently been recognized. They allow reversible changes in gene expression that are responsible for tumor initiation and progression, and escape from immune surveillance or drug resistance. On the other hand, their reversibility provides an opportunity for their pharmaceutical targeting. However, a deep understanding of epigenetic landscapes that drive PDAC, their interactions with genetic changes, and influences of immune system will pave the way for the discovery of new markers and will guide development of combination therapies for personalized treatment.

## 2. PDAC Genomics and its Clinical Relevance

Whole-genome and transcriptome sequencing studies have provided key insights into the molecular pathogenesis of PDAC via the identification of complex and heterogeneous mutational profiles. These affect the core molecular pathways including DNA repair, RNA processing, axonal guidance, and cell cycle and chromatin regulation. Genome-wide screening methods have shown that *KRAS, SMAD4, TP53*, and *CDKN2A* are the four most frequently altered genes in pancreatic adenocarcinoma [13,14,15,16]. *KRAS* is the most frequently mutated gene in PDAC (95%) and plays a central role in PDAC cancer cell growth and survival [17,18,19]. These tumors were classified into four subtypes with potential clinical relevance based on chromosomal structure and copy number variation (CNV) analysis of 100 PDAC cases. The subtypes were classified as stable, locally rearranged, scattered, and unstable. The genomes of stable tumors contained ≤50 structural variation events, whereas locally rearranged tumors displayed a significant focal event on one or two chromosomes. The scattered tumor class exhibited less than 200 structural variations. Finally, unstable tumors showed a large number (>200; maximum of 558) of structural variation events representing genomic instability [20].

Genomic and transcriptomic analysis of 456 PDACs identified 32 recurrently mutated genes and defined four subtypes with differences in the molecular evolution of the disease (squamous, progenitor, immunogenic, and aberrantly differentiated endocrine exocrine (called ADEX) tumors). Moreover, the subtypes were characterized by different mutational landscapes, tumor histopathological features, and prognosis. The classification of diagnosed patients into one of these four subtypes may have a prognostic value and provide a more personalized treatment [21]. A comprehensive genetic analysis that targeted all exons and selected introns of 410 cancer-associated genes was performed with tumor samples from 336 pancreatic carcinoma patients. This study identified potentially actionable findings in 26% of cases. In accordance with other studies, the results demonstrated frequent genetic alterations in key signaling pathways, including Notch signaling, chromatin remodeling, DNA repair, cell cycle, RNA processing, WNT and TGF beta (TGF-β) signaling, and KRAS activation. However, according to the authors, the therapeutic implications of the molecular results appeared to be limited, as only 1% of patients received a matched therapy based upon the sequencing results. This included patients with germline BRCA1/2 mutations who underwent a surgical resection followed by adjuvant platinum-based therapy, with no evidence of disease recurrence after treatment [22]. Molecular testing for BRCA mutations followed by treatment with cisplatin and a standard chemotherapy backbone should be considered, due to the prognostic benefit from DNA crosslinking agents in BRCA-positive cases [23,24,25]. Poly (ADP-ribose) polymerase (PARP) inhibitors have shown a benefit on maintenance treatment in these groups of patients [26]. At the same time, germline alterations in other DNA damage repair (DDR) genes such as *MDC1, ATR*, and *ATM* may represent a biomarker to define patients who will also benefit from the treatment with agents that target a defective DDR [27,28,29]. In fact, an estimated 4–10% of pancreatic cancers have a familial background [30], and many of these cases have germline mutations in DNA repair genes [31].

More recently, a retrospective meta-analysis of gene expression profiles from 1200 PDAC patients identified six molecular and clinically distinct subtypes, which describes a more complete picture of PDAC heterogeneity. Moreover, the clinical outcome for each subtype was predicted. A large cohort with high statistical power provided a deep learning-based classification system containing 160 subtype-specific markers, which better stratifies PDAC patients for personalized treatments [32].

## 3. Epigenetic Changes Associated with PDAC

Complex mechanisms underlying the development and progression of PDAC include the interaction between genomic, epigenomic, and signaling pathway alterations. The extensive research in genetics and genome-wide expression patterns indicates that genetic changes are critical for PDAC initiation and early progression. However, epigenomic studies revealed that epigenetic alterations in oncogenes and tumor suppressor genes affected tumor progression and were associated with survival [33]. Epigenetic changes are heritable modifications of DNA or chromatin structures, which influence gene expression without altering the DNA sequence [34]. Epigenomics of PDAC studies the epigenetic changes across multiple genes or the entire genome. DNA methylation, post-translational modifications of histone proteins, and non-coding RNAs are the main epigenetic mechanisms that can influence gene expression. They have been confirmed as important contributors to PDAC development and progression [35,36,37].

### 3.1. DNA Methylation

Epigenetic alterations have already been observed in early PDAC lesions, i.e., PanIN-1A. Thus, DNA methylation may represent an early event during tumorigenesis of PDAC [38]. Silencing of a gene by DNA methylation is catalyzed by the addition of a methyl group by DNA methyltransferases (DNMTs) to the 5′ carbon of the cytosine pyrimidine ring within CpG islands. These are preferentially located in regulatory regions of genes [39]. DNA methyltransferase 1 (DNMT1) is considered to be the key enzyme for the maintenance of parental DNA methylation patterns following replication, while de novo DNA methylation is catalyzed by DNMT3A and DNMT3B [40]. DNMT1 overexpression was observed in approximately 80% of pancreatic carcinomas [41], where many promoters of key tumor suppressor genes (TSGs) were hypermethylated [42]. Overexpression of DNMT1 was reported to be responsible for silencing key tumor suppressor genes such as *p16, PENK*, and *RASSF1* [43]. Moreover, several studies on PDAC have reported an increase in non-specific and targeted gene-specific 5-hydroxymethylation. An increase of 5-hmC, which is the metabolic product of 5-mC, has been described mainly in enhancers of key oncogenes such as *MYC*, *KRAS*, *VEGFA*, and *BRD4* [44]. Therefore, 5-hmC is believed to play an important regulatory role in PDAC pathogenesis and progression [45]. Interestingly, genome-wide comprehensive methylation profiling of PDAC patient-derived xenografts (PDXs) demonstrated that PDAC stem cells (CSCs) that express high levels of DNMT1 lost the stemness phenotype upon pharmacologic or genetic DNMT1 inhibition [46]. Furthermore, genomic instability due to hypomethylation (aberrant loss of methylation) was also found in PDAC. Subsequent overexpression of the genes *SERPINB5*, *CLDN4*, *SFN*, *LCN2*, *TFF2*, and *S100P* was found compared to normal pancreatic ductal cells. This led to numerous alterations in cell cycle progression, proliferation, differentiation, or adhesion [6].

### 3.2. Histone Modifications

Chromatin condensation, nucleosome positioning, and transcription machinery access to DNA are orchestrated by post-translational modifications of histone proteins. At least nine different types of histone modifications have been discovered to date. The most frequently studied are acetylation, methylation, phosphorylation, and ubiquitylation. Together, histone modifications regulate the transcriptional state of the local genomic region [47]. These modifications are deposited by enzymes called “writers”, hydrolyzed or degraded by “erasers”, and recognized and bound by “readers”. Histone acetylation and deacetylation present key epigenetic signals that regulate gene expression. The enzymes that mediate these reactions are histone acetyltransferases (HATs) such as CBP, p300, and PCAF, which transfer an acetyl group to lysine residues within histones and non-histone proteins. This process leads to chromatin relaxation and may facilitate the accessibility of the DNA to transcription factors and whole transcriptional machinery. On the other hand, the reverse reaction, histone deacetylation mediated by histone deacetylases (HDACs), is associated with gene repression. However, the balance between histone acetylation and deacetylation is very dynamic and both groups of enzymes could bind to gene promoters through physical interaction with sequence-specific transcription factors, regardless of their transcriptional activity [48]. While HDACs control various key oncogenic features in PDAC, HDAC inhibition has been studied as a therapeutic strategy. Recent clinical trials in PDAC evaluated the safety and efficacy of HDAC inhibitors (HDACis) in combination with small-molecule inhibitors or chemotherapeutic agents. Some results have shown a synergism of the gemcitabine/HDACi combination, but the majority of studies were restricted by a limited number of patients with distinct histological PDAC subgroups [49].

### 3.3. microRNAs

Deregulation of microRNAs (miRNAs) contributes to PDAC carcinogenesis by promoting the expression of proto-oncogenes or inhibiting the expression of tumor suppressor genes [50], among others. They represent a highly conserved subfamily of non-coding RNA molecules of 18–24-nucleotides that regulate the transcription of target messenger RNAs [51]. They are implicated in the regulation of cellular differentiation, proliferation, and apoptosis. As previously reported, numerous miRNAs, including miR-200, miR-34, miR-21, miR-155, miR-221, and miR-222, are aberrantly expressed in PDAC [52,53,54]. The study by Hong and Park of miRNA expression profiling of PDAC specimens revealed the five most upregulated miRNAs as miRNA-21, miRNA-196a, miRNA-27a, miRNA-146a, and miRNA-200a. On the other hand, miRNA-96, miRNA-217, miRNA-141, miRNA-20a, and miRNA-29c were the most downregulated in the tested cohort [55]. Specific miRNA expression profiles may correlate with different stages of PDAC and are potential biomarkers, prognostic markers, and clinical targets [56]. The stability of miRNA in blood allows plasmatic levels of specific miRNA to be linked with stage, survival rate, or disease aggressiveness [57,58]. The detection of miRNAs in liquid biopsies is very relevant and useful in PDAC, where primary tumor biopsies are very scarce. In this context, there was a significant correlation between plasma miR-221 levels and clinicopathological outcomes among 47 patients with pancreatic carcinomas. Thus, plasma miR-221 concentrations determined before surgery may be a useful clinical biomarker to predict distant metastasis and tumor resectability. The plasmatic level of miR-221 also showed differences between cancer patients and patients with pancreatic benign tumors, suggesting its potential role as a biomarker for early cancer detection [59]. Recently, a comprehensive expression analysis of miR-200a, miR200b, miR-200c, miR-141, miR-429, and miR-205 identified significant changes in expression in pancreatic tumors and PDXs. The results indicate that members of the miR-200 family, in particular miR-429, may act as tumor suppressor genes in PDAC [60]. Moreover, miRNAs could be also useful biomarkers for prognosis and treatment response, since they contribute to chemoresistance mechanisms [56].

## 4. Epigenomic Landscapes and Molecular Tumor Heterogeneity

PDAC tumor heterogeneity, which leads to many cell subtypes within the primary tumor, introduces significant challenges in the design of effective treatment strategies. Studies have shown epigenetic changes that regulate the malignant phenotype by transcriptomic alterations. Lomberk et al. provided the first knowledge on specific epigenomic landscapes linked to PDAC tumor heterogeneity in 24 human samples grown as PDXs [61]. This multi-parametric integrative analysis studying chromatin states, DNA methylation, and gene expression led to a definition of two molecularly distinct PDAC subtypes, namely basal and classical, differing in clinical outcomes. For both subtypes, the state of promoters, enhancers, super-enhancers, euchromatic, heterochromatic regions, and the binding sites of multiple protein targets were characterized. According to the results, PDAC biology seemed to be regulated by specific combinations of histone marks and DNA methylation in genes, involved in major carcinogenic pathways. These included RB, TP53, and other cell cycle checkpoints involved in proliferation and apoptotic control, i.e., ErbB, IGF, RAS, mTOR, and TGF signaling pathways, and cell adhesion molecules (cadherins and integrins). The functional analysis of the more aggressive, basal PDAC subtype identified enhanced genes implicated in signal transduction pathways with a strong oncogenic potential (e.g., ErbB/EGFR, PI3K-AKT, Hippo, and Wnt), EMT (e.g., TGF-β) as well as the deregulation of cell differentiation, proliferation, and apoptosis (e.g., YAP1, HEY1, MYC, and E2F7). On the other hand, the epigenomic landscape of classical PDAC subtype showed activation of genes involved mainly in pancreatic development (e.g., PDX1, BMP2, GATA6, SHH), metabolic processes (e.g., HKDC1, FBP1), and Ras signaling (e.g., KITLG, RASA3). Interestingly, MET inactivation by small interfering RNA (siRNA) led to a regression of the aggressive basal subtype to a more benign form that supports the future testing of anti-MET therapies in PDAC [61].

The recent study of reprogrammed primary PDAC cultures using episomal vectors confirmed that epigenetic alterations accounted for the tumorigenicity and aggressiveness of PDAC, at least in part. Reprogrammed cells, which were driven by the cells’ enhanced differentiation and rapid loss of stemness, were functionally distinct from parental PDAC cells. This resulted in a drastically reduced tumorigenicity in vitro and in vivo [62]. These findings strongly support the use of epigenetic modulators (epi-drugs) as a suitable approach to increase survival in PDAC patients.

## 5. Epigenetics and Epithelial–Mesenchymal Transition in PDAC

**Figure 2:**
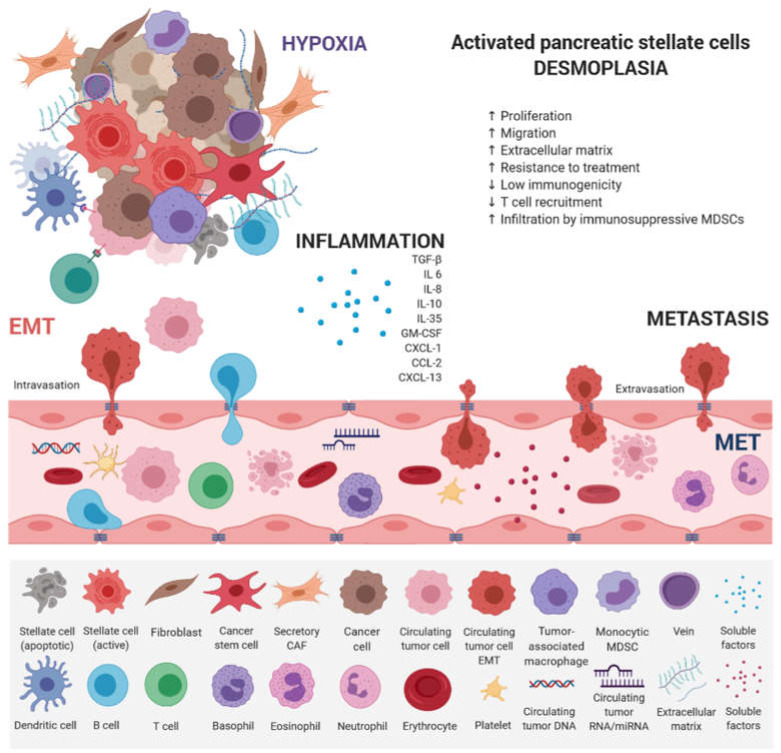
Main factors that influence PDAC progression. PDAC consists of a minority of malignant cells within a microenvironment composed of an extracellular matrix (ECM) and a plethora of hematopoietic cells. Pancreatic stellate cells activated from the quiescent state by intrinsic and extrinsic stimuli (e.g., mechanical injury or chronic inflammation) play a key role. Once activated, they secrete an abundant amount of ECM proteins composed of hyaluronan, laminin, fibronectin, and collagens. By secreting ECM proteins, cytokines, and chemokines, they actively recruit monocytes and CD4 regulatory T cells (Tregs). Hematopoietic cells are predominantly of the myeloid lineage, with both granulocytic and monocytic myeloid-derived suppressor cells (MDSCs), as well as tumor-associated macrophages (TAMs). All contribute to local immunosuppression. *KRAS* mutation is an early oncogenic event that drives the recruitment of B cells early in the development of PDAC, which can exert immunosuppressive effects. Therefore, T cells, both CD4+ and CD8+, are not primed against PDAC antigens and are not excluded from tumors. The paucity of blood vessels leads to high levels of hypoxia in the interior of the tumor. Paracrine interactions between inflammatory cells and hypoxia stimuli induce EMT, which promotes circulating tumor cell (CTC) formation and endows differentiated normal and cancer cells with stem cell properties. They are characterized by a high expression of drug efflux pumps including multi-drug resistance genes, protecting them from chemotherapeutic reagents thus increasing their metastatic potential.

EMT has emerged as a clinically relevant regulator of tumor cell spread to distant organs [63]. Early metastasis of PDAC results from dynamic gene expression changes, allowing epithelial tumor cells to acquire mesenchymal, fibroblast-like properties, increased migratory capacity, invasiveness, and resistance to therapy [64]. A cross-talk between individual counterparts of tumor microenvironment and innate and adaptive immune cells leads to PDAC progression (Figure 2).

This dedifferentiation process is associated with a loss of functional epithelial cell markers, such as E-cadherin, and increased expression of mesenchymal markers [65]. Stem cell-associated marker expression of CD24+, CD44+, and CD133+ cells was found to correlate with EMT in PDAC [66]. TGF-β, TNF-α, as well as Notch, Hedgehog, and Wnt molecular pathways have been shown to induce EMT by the upregulation of Snail, Twist, and Zeb1 transcription factors, which destroys the tight junctions and degrades adhesion molecules [67,68,69,70,71,72,73]. Reversibility of EMT-related changes via epigenetic regulation allows the tumor cell to respond to various external and internal stimuli. Decreased expression of the *CDH1* gene, which encodes the glycoprotein E-cadherin, is considered the hallmark of EMT, characterized by the dedifferentiation of epithelial cells and a loss of intercellular junctions [74]. Loss of E-cadherin-mediated cell–cell adhesion was associated with higher tumor invasiveness and increased metastatic potential [75]. This occurs in 42–60% of PDAC tumors and shows a strong correlation with metastasis to remote organs. However, a mechanism of *CDH1* downregulation in most tumors is unknown. In fact, a decreased expression of the *CDH1* gene is rarely induced by mutation or loss of heterozygosity. According to the published data, hypermethylation of the gene promoter region and interaction with the EMT-inducing transcription factors Slug, Snail, and Twist1 may explain the decreased *CDH1* gene expression [76,77]. Moreover, von Burstin et al. demonstrated a downregulation of E-cadherin in metastatic PDAC cells mediated by a repressor complex, containing Snail and HDACs. The Snail protein binds directly to the *CDH1* promoter, resulting in the inhibition of *CDH1* expression, and Snail mRNA overexpression has been shown to correlate with the metastatic potential of cells [78]. These results were in accordance with previous findings showing an association between EMT and HDAC-mediated transcription control [79]. Several miRNAs were also identified as modulators of EMT, mainly the members of the miR-200 family and miR-205 [80], and their participation in PDAC tumors was also confirmed [81]. The role of epigenetic factors in EMT during malignant progression in PDAC was also confirmed by the epigenetic silencing of FOXA1/2 transcriptions factors, which were determined to be effective antagonists of EMT by promoter hypermethylation [82].

## 6. Early Detection—Promises and Challenges of Liquid Biopsy

Early diagnosis in parallel with the search for new treatments is the only strategy that can help to alleviate a dismal prognosis of PDAC patients. Liquid biopsies are of particular interest as they allow the non-invasive identification of tumor-associated biomarkers in body fluids when clinical symptoms are still absent and conventional diagnostic tools do not offer sufficient analytical sensitivity. The liquid biopsy is of the utmost importance in PDAC as fresh tissue is scarce. Moreover, these biopsies may be used for disease monitoring and the detection of relapses, which remains as high as 80%, despite radical surgical resection [83]. In PDAC, several studies have focused on the analysis of CTCs, circulating tumor DNA (ctDNA), or membrane-bound extracellular vesicles (EVs), specifically exosomes. Diagnostic performance of individual methods differed depending on disease stage, while a significant prognostic value was demonstrated in 50–70% of clinical studies, irrespective of the liquid biopsy type [84]. The only blood-based marker currently available in clinical settings is the carbohydrate antigen 19-9 (CA 19-9), which displays a low sensitivity and specificity for early stages to be reliably used for PDAC diagnostics [85].

CTCs were associated with poor PDAC patient prognosis and an increased risk of relapse. However, CTC-based diagnosis was proven to be highly specific, since the false-positive rate was reported as close to zero in the majority of studies. The sensitivity was influenced by the rarity of CTCs, which are not efficiently captured or enriched by currently available methods [86]. The results of the main clinical pilot studies that assessed the performance of CTC detection in the diagnosis and prognosis of PDAC were recently reported [84].

*KRAS* mutation detection in ctDNA in plasma and serum is one of the most frequently used liquid biopsy approaches for PDAC [84]. Highly fragmented ctDNA (160–180 bp) originates from apoptotic or necrotic cells but can be also actively released by the tumor cells in exosomes [87]. Digital droplet PCR (ddPCR) has been proven as one of the most suitable methods for rare event detection. Its analytical sensitivity is 0.01% in comparison to direct sequencing (10–30%) or next-generation sequencing (10%) [88]. The main studies that have investigated the role of ctDNA in the diagnosis and/or prognosis of PDAC were reviewed recently [84]. Based on the published results, it is highly likely that the presence of ctDNA not only represents a valuable diagnostic tool but it also plays a role in PDAC prognosis. Although tumor-associated DNA methylation changes emerged recently as a promising approach for cancer risk assessment and monitoring, they have been rarely assessed in PDAC [89,90]. Nevertheless, a recent study has shown the value of *SPARC* and *NPTX2* hypermethylation in cfDNA, which were associated with metastasis and poor survival and also differentiated PDAC from chronic pancreatitis [91].

Exosomes, similarly to other EVs, have the ability to relay signals between cells. There is currently no universal method for extracellular vesicle isolation from body fluids, although ultracentrifugation or kit-based precipitation have been reported for PDAC, focusing on PDAC-specific molecular characterization of the enriched samples [84]. However, this expanding area of research needs a robust assessment of the sensitivity and specificity, although it holds great promise.

Non-coding RNAs, mainly miRNAs that are discussed in detail above, have been studied in the context of PDAC. In fact, several distinct miR signatures have been reported with different sensitivities and specificities, such as miR-17-5p, miR-191, miR-451a, miR-21, miR-1246, miR-4644, miR-3976, miR-4306, and others [84]. Several of them showed a better diagnostic accuracy than CA19-9 for early-stage cancers [92]. Moreover, plasma levels of miR-21 determined before surgery was a sensitive biomarker and an independent prognostic factor in surgical settings [93].

Despite these promises, there are still many challenges that hamper the routine use of liquid biopsies as a clinical tool for the non-invasive diagnosis and prognosis of PDAC. Beyond mutational burden, sensitive epigenetic screening panels may allow an effective identification of early cancer stages, disease monitoring, and detection of disease relapse. The identification of aberrant patterns using methylation signatures opens up novel treatment options that include epigenetic therapeutic strategies. A combination of innovative technical approaches that focus on epigenomic and proteomic profiles will allow the application of a multi-biomarker approach, overcoming the limitations of single biomarkers, and ultimately benefiting PDAC patients.

## 7. Search for New Epigenetic Treatments in PDAC

Although significant improvements have been achieved in the treatment of many solid tumors, PDAC remains poorly addressed. Besides the lack of reliable early biomarkers, the main causes include dense and diffused desmoplastic stroma, tumor heterogeneity with a broad mutational landscape, followed by overactivation of many signaling pathways that accumulate during disease progression. More than 70% of patients do not respond to the currently applied regimens and conventional treatment, chemotherapy, and radiotherapy offer marginal benefits. Usually, they are used as a first-line treatment for non-resectable disease [94]. Single-agent therapies using gemcitabine and its combinations, multidrug regimens such as FOLFIRINOX (the combination of folinic acid, 5-fluorouracil, irinotecan, oxaliplatin) and targeted approaches, have not improved patient survival [95]. Moreover, the failure of the first-line chemotherapy leads to an acceleration of growth, conducive to a resistant and metastatic tumor [96]. Immunotherapy has achieved a compelling success (mainly immune checkpoint inhibitors) in several cancers, including melanoma, urothelial, head and neck, or lung cancer [97]. Regarding PDAC, all immunotherapeutic efforts have failed so far. However, a combination of immunotherapy with epigenetic therapy, recently reviewed by Topper et al., represents a promising trend to bring the improvements in treatment of PDAC patients [98].

**Figure 3:**
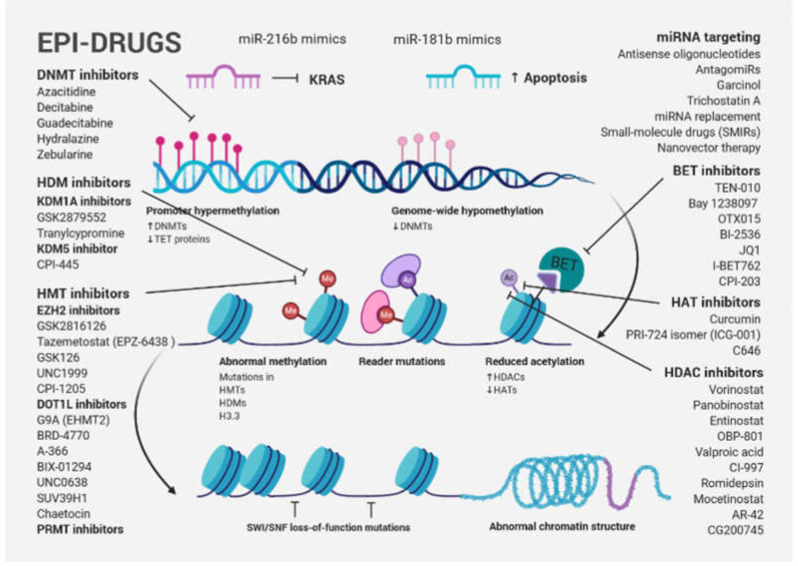
Schematic presentation of cancer-associated epigenetic modification abnormalities, including a brief overview of epi-drugs used in preclinical studies and clinical trials for PDAC treatment. Gene-specific hypermethylation accompanied by global DNA hypomethylation, frequent mutation of epigenetic modifiers and histone proteins, as well as abnormal histone modification patterns are typical cancer-associated epigenetic changes. A large number of the first-, second- and third-generation inhibitors, targeting epigenetic modifications, were used in preclinical studies and clinical trials. Although epi-drug monotherapies were not successful, combination therapy targeting diverse chromatin regulators or a biomarker-directed approach that combines a new generation of epi-drugs with traditional approaches currently represent the most promising concept for PDAC.

One of the new avenues in solid tumor clinical investigation is the potential of epi-drugs to modulate the sensitivity of tumors to other anticancer agents. Primary and acquired resistance to treatment, which is a hallmark of PDAC, may be a consequence of dynamic and reversible epigenetic changes that confer a fitness advantage to tumor cells, known as epigenetic plasticity [99]. DNMT inhibitors (DNMTis) and HDACis were originally approved by the Food and Drug Administration (FDA) and European Medicines Agency (EMA) for certain hematological malignancies. Great hopes were also placed in them for the treatment of solid tumors. However, despite robust preclinical evidence, first-generation epi-drugs have shown a low efficacy, such as the DNMTis azacytidine and decitabine and the HDACis vorinostat and romidepsin [100]. One possible explanation for their failure is that more differentiated solid tumors with terminally differentiated cells have a reduced capability for epigenetic reprogramming. Although the second-generation of epi-drugs (the DNMTis zebularine and guadecitabine and the HDACis belinostat, panobinostat, tucidinostat (chidamide), and valproic acid) have shown improved chemical properties, limited efficacy and significant side effects have been observed [100]. Recently, a new generation of selective epi-drugs is being developed; they are more specific for their targets and are entering clinical testing (Figure 3).

Bromodomain and extra-terminal domain inhibitors (BETis), histone methyltransferase inhibitors (HMTis), including enhancer of zeste homologue 2 inhibitors (EZH2is) and DOT1-like histone-lysine methyltransferase inhibitors (DOT1Lis), histone demethylase inhibitors (HDMis) such as lysine-specific histone demethylase 1A inhibitors (KDM1Ais) and protein arginine methyltransferase inhibitors (PRMTis), among others, are included in this group [100].

To reverse resistance and sensitize cancer cells to treatment, epi-drugs were tested in combination with other anticancer therapies, such as genotoxic and/or cytotoxic chemo- and radiotherapy, targeted therapies, as well as hormone and immunotherapies. Drug resistance in PDAC is mediated by a pronounced cell plasticity (the ability to switch between different phenotypic states), allowing individual cellular clones to evade therapy [49]. HAT inhibitors (HATis), ICG-001 and C646, were found to reduce tumorigenicity of PDAC in preclinical cell models [101,102,103] and the PRI-724 isomer (ICG-001) is already used in a clinical trial for advanced and metastatic pancreatic adenocarcinoma (NCT01764477). However, little progress has been made to evaluate the benefit of HAT inhibition in the treatment of PDAC patients. ChIP-seq data from seven widely used PDAC cell lines corresponding to low (CAPAN1, CAPAN2, CFPAC1, HFPAC1) and high (PANC1, MiaPaCa2, PT45P1) grade groups showed that low-grade PDAC cells had an increased number of super-enhancer domains, whereas both groups had a similar number of broad H3K4me3 (a mark of active promoter regions). In the high-grade model, PANC1, treatment with either ICG-001 or C646 resulted in dramatic effects on the epigenome with hundreds of regions showing a differential enrichment of H3K27ac or H3K4me3. Interestingly, both drugs targeted similar super-enhancers, causing a reduction in histone acetylation levels near genes involved in PDAC and other solid cancers [104]. Several epi-drugs were tested in phase I/II PDAC clinical trials, namely EZH2i EPZ-6438, GSK2816126, HATi Curcumin, BETi TEN-010, Bay 1238097, OTX015 and BI-2536, and the HDACis vorinostat, panobinostat, entinostat, OBP-801, valproic acid, CI-997, romidepsin, and mocetinostat. Although epi-drug monotherapies were not successful, combination therapy that targets diverse chromatin regulators (e.g., BETis and HDACis) currently represent the most promising concept for PDAC [49]. Genes involved in cell cycle regulation (*CDKN1A*, *XRCC6*), inhibition of epithelial differentiation (*GATA4*, *GATA6*, *DAB2*, *MUC2*), apoptosis (*TP53*), as well as EMT and metastasis (*CDH1*, *CXCR4*, or miR-203) are HDAC targets.

The right combination, dosing, and sequence may improve the efficacy and tolerability of epi-drugs, in combination with other types of anticancer therapies. The identification of precise biomarkers for patient stratification and subsequent implementation of personalized strategies in clinical trials represent an urgent requirement for successful PDAC treatment.

## 8. Future Directions and Clinical Implications

PDAC remains a devastating disease, despite extensive research efforts focused on the discovery of early diagnostic biomarkers and efficient therapeutic approaches, showing a high resistance to treatment that significantly contributes to poor survival. Bearing in mind its incidence and mortality rate estimates, we are in desperate need of novel biomarkers and treatment options. The success of epi-drugs in hematological malignancies and the robust body of evidence supporting the hypothesis about their ability to synergize with other anti-cancer drugs, sensitizing resistant tumor cells in preclinical models, have encouraged the search for these agents suitable for the future clinical management of PDAC. However, a lack of predictive markers for patient selection and a high degree of tumor heterogeneity, which results in distinct subtype-specific epigenetic landscapes of PDAC tumors, means that old generation epi-drugs are not specific enough. The success of PDAC treatment will be determined by the integration of genomic, epigenomic, and transcriptomic data into a biomarker-driven approach, combining new generations of epi-drugs with traditional and new therapies. Finally, overcoming the current methodological obstacles that hamper the large-scale use of the liquid biopsy in clinical practice will hopefully, in the near future, allow early disease detection and reliable monitoring and provide information on therapeutic targets and resistance mechanisms for the management of individual patients.

## Abbreviations

Ac	Acetylation
ADEX	Abberantly differentiated endocrine exocrine
ATM	Ataxia telangiectasia mutated
ATR	Ataxia telangiectasia mutated and Rad3 related
BET	Bromodomain and extra-terminal motif
BMP2	Bone morphogenetic protein 2
BRD4	Bromodomain-containing 4
CAF	Cancer-associated fibroblast
CBP	CREB-binding protein
CCL-2	Chemokine (C-C motif) ligand 2
CDH1	Cadherin 1
CDKN2A	Cyclin-dependent kinase inhibitor 2A
CTC	Circulating tumor cell
CXCL-1	Chemokine (C-X-C motif) ligand 1
CXCL-13	Chemokine (C-X-C motif) ligand 13
CXCR4	C-X-C motif chemokine receptor 4
DAB2	DAB adaptor protein 2
DNA	Deoxyribonucleic acid
DNMT	DNA methyltransferase
DOT1Li	Histone H3K79 methyltransferase inhibitor
ECM	Extracellular matrix
EGFR	Epidermal growth factor receptor
EHMT2	Histone-lysine N-methyltransferase known as G9A
EMT	Epithelial–mesenchymal transition
EZH2	Enhancer of zeste homolog
FBP1	Fructose-bisphosphatase 1
FOXA1/2	Forkhead box A1/2
HAT	Histone acetyltransferase
HDAC	Histone deacetylase
HDM	Histone demethylase
HKDC1	Hexokinase domain-containing 1
HMT	Histone methyltransferase
H3.3	Histone 3.3
IGF	Insulin-like growth factor
IL	Interleukin
KDM1Ai	Lysine-specific histone demethylase 1A inhibitor
LCN2	Lipocalin-2
MDC1	Mediator of DNA damage checkpoint 1
MDSC	Myeloid-derived suppressor cell
Me	Methylation
MET	Mesenchymal–epithelial transition
miRNA	microRNA
mTOR	Mammalian (or Mechanistic) target of rapamycin
MUC2	Mucin 2
NPTX2	Neuronal pentraxin 2
PanIN	Pancreatic intraepithelial neoplasia
PARP	Poly (ADP-ribose) polymerase
PCAF	P300/CBP-associated factor
PDAC	Pancreatic ductal adenocarcinoma
PDX	Patient-derived xenograft
PDX1	Pancreatic and duodenal homeobox 1
PENK	Proenkephalin
PI3K	Phosphatidylinositol 3-kinase
PRMT	Protein arginine methyltransferase
PSC	Pancreatic stellate cell
RASSF1	Ras association domain family member 1
RB	Retinoblastoma
RNA	Ribonucleic acid
S100P	S100 calcium-binding protein P
SERPINB5	Serpin peptidase inhibitor, clade B, member 5
SFN	Stratifin
SHH	Sonic hedgehog
SPARC	Secreted protein acidic and cysteine rich
SUV39H1	Histone-lysine N-methyltransferase
SWI/SNF	ATP-dependent chromatin remodeling complex
TAM	Tumor-associated macrophage
TET	Ten-eleven translocation protein
TFF2	Trefoil factor 2
TGF-β	Transforming growth factor-beta
VEGFA	Vascular endothelial growth factor A
